# Heteroplasmic mitochondrial DNA mutations in frontotemporal lobar degeneration

**DOI:** 10.1007/s00401-022-02423-6

**Published:** 2022-04-30

**Authors:** Yu Nie, Alexander Murley, Zoe Golder, James B. Rowe, Kieren Allinson, Patrick F. Chinnery

**Affiliations:** 1 Department of Clinical Neurosciences, School of Clinical Medicine, University of Cambridge, Cambridge Biomedical Campus, Cambridge, UK; 2 Medical Research Council Mitochondrial Biology Unit, University of Cambridge, Cambridge Biomedical Campus, Cambridge, UK; 3 Department of Neuropathology, Cambridge University Hospitals NHS Foundation Trust, Cambridge, UK; 4 Department of Clinical Neurosciences, University Neurology Unit, Level 5, A Block, Cambridge Biomedical Campus, Box 165, Cambridge, CB2 0QQ UK

## Abstract

Frontotemporal lobar degeneration (FTLD) is a common cause of young onset dementia and is characterised by focal neuropathology. The reasons for the regional neuronal vulnerability are not known. Mitochondrial mechanisms have been implicated in the pathogenesis of FTLD, raising the possibility that frontotemporal regional mutations of mitochondrial DNA (mtDNA) are contributory causes. Here we applied dual sequencing of the entire mtDNA at high depth to identify high-fidelity single nucleotide variants (mtSNVs) and mtDNA rearrangements in *post mortem* brain tissue of people affected by FTLD and age-matched controls. Both mtSNVs and mtDNA rearrangements were elevated in the temporal lobe, with the greatest burden seen in FTLD. mtSNVs found in multiple brain regions also reached a higher heteroplasmy levels in the temporal lobe. The temporal lobe of people with FTLD had a higher burden of ribosomal gene variants predicted to affect intra-mitochondrial protein synthesis, and a higher proportion of missense variants in genes coding for respiratory chain subunits. In conclusion, heteroplasmic mtDNA variants predicted to affect oxidative phosphorylation are enriched in FTLD temporal lobe, and thus may contribute to the regional vulnerability in pathogenesis.

**Supplementary Information:**

The online version contains supplementary material available at 10.1007/s00401-022-02423-6.

## Introduction

Frontotemporal lobar degeneration (FTLD) is a common cause of young-onset dementia with prevalence of ~ 3–5/100,000 [1]. The clinical syndromes associated FTLD typically present in mid-to-late adult life with cognitive and movement disorders and the clinical diagnoses of behavioural form of frontotemporal dementia, the primary progressive aphasias, progressive supranuclear palsy, corticobasal syndrome and motor neuron disease (amyotrophic lateral sclerosis) [2]. Brain imaging and *post mortem* data reveal strikingly regional pathology, from which FTLD is named. There are nuclear and cytoplasmic neuronal protein inclusions associated with the proliferation of glia leading to frontal and temporal lobar degeneration. The molecular pathophysiology of FTLD is complex, involving multiple parallel and often interacting pathways centred around aberrant cytoplasmic protein folding and aggregation leading to inclusions of Tau protein, TAR DNA binding protein 43 (TDP43) or Fused in Sarcoma protein (FUS) [3]. There is also impairment of autophagy, which is a highly energy-dependent process [4]. Genetic factors play a prominent role in the pathogenesis of FTLD, with single high-penetrance variants in *MAPT*, *GRN*, *C9ORF72* found in ~ 20% of affected individuals; other autosomal dominant genes in 10–20%, and multiple risk loci identified through genome wide association studies [5]. Although important, inherited nuclear genetic variants cannot account alone for the strikingly regional nature of FTLD pathology, implicating other mechanisms in the regional neuronal vulnerability.

Mutations in *CHCHD10* found in FTD-ALS suggest that mitochondrial energy metabolism can play a role in the pathogenesis of FTLD. *CHCHD10* codes for a poorly understood mitochondrial protein that interacts with the mitochondrial contact site and organising system complex (MICOS) leading to the formation of multiple deletions of mitochondrial DNA (mtDNA) [6]. The mtDNA codes for 13 essential respiratory chain proteins that are required for the adenosine triphosphate (ATP) synthesis by aerobic metabolism. Unlike nuclear DNA, neurons and glia contain thousands of copies of mtDNA, and recent mutations only affect a proportion of these molecules (known as heteroplasmy) [7]. Heteroplasmy levels vary from cell to cell, raising the hypothesis that regional pathology within the brain reflects the regional variance of pathogenic mtDNA variants. We tested this hypothesis by sequencing the entire mtDNA at very high depth to measure the number (burden) and heteroplasmy level of single nucleotide variants (mtSNVs) and deletions (mtDels) and duplications (mtDups) across the entire mitochondrial genome. We sequenced tissue from three brain regions sampled *post mortem* from people who were affected by FTLD, and age matched controls without dementia. Our findings implicate mtDNA mutations in the regional pathology of the disorder.

## Methods

### Study participants

We studied left temporal lobe (superior temporal gyrus cortex), left occipital lobe (striate cortex) and medulla (tegmentum at the level of the open fourth ventricle). We also sampled brains *post mortem* from age-matched controls with no clinical or neuropathological evidence of neurodegenerative disease (Table 1). Sixteen cases of autopsy confirmed FTLD (frontotemporal lobar degeneration) were selected. This included thirteen cases of FTLD-TDP (two of which were linked to C9orf72), two cases of FTLD-U (with ubiquitin and p62-positive inclusions, negative for tau, beta-amyloid and alpha-synuclein) and a case of FTLD-tau with classical Pick’s disease. In the pathology group, the mean age at death was 68 years (range 46–90 years) and the mean *post mortem* interval (PMI) was 34.7 h (range 5–81 h). Fifteen control cases were selected. These had no significant neurodegenerative disease on autopsy examination. The mean age in the control group was 67.8 years (range 35–95 years) and the mean *post mortem* interval was 50 h (range 12–89 h). There was no significant difference in the age (Student’s *t*-test, *p* = 0.95), PMI (Student’s *t*-test, *p* = 0.06) or male/female ratio (Fisher's exact test, *p* = 1) between cases and controls.

**Table 1:** Neuropathological characteristics, *post-mortem* interval, sex and age at death for the 31 frozen brains

Case	Post-mortem diagnosis	FTLD subtype	Sex	Age	TDP-43	Braak stage (NFTs)	Braak stage (LP)	CERAD score (NPs)	Thal stage	PMI (h)
P1	FTLD-TDP	A	M	60	+	0	0	0	0	13
P2	FTLD-TDP	A	M	72	+	2	0	0	0	10
P3	FTLD-TDP	B	M	70	+	2	0	0	1	29
P4	FTLD-TDP	B	F	67	+	2	0	0	0	36
P5	FTLD-FUS	NIFID	F	46	–	0	0	0	0	15
P6	FTLD-TDP	A	F	71	+	0	0	0	0	39
P7	FTLD-TDP	C	M	77	+	0	0	0	0	5
P8	FTLD-TDP	C	F	77	+	0	0	0	0	43
P9	FTLD-TDP	A	M	70	+	2	0	1	1	35
P10	FTLD-TDP	C	M	64	+	0	0	0	1	74
P11	FTLD-TDP	A	F	79	+	3	0	0	0	30
P12	FTLD-UPS	N/A	M	50	–	0	0	0	0	28
P13	FTLD-TDP	C	M	90	+	0	0	0	0	81
P14	FTLD-TDP with ALS (C9orf72)	B	F	65	+	2	0	0	0	49
P15	FTLD-TDP with ALS (C9orf72)	B	M	61	+	1	0	0	0	28
P16	FTLD-tau (Pick’s disease)	N/A	F	70	–	0	0	0	0	40
C1	None	N/A	M	61	–	0	0	0	0	55
C2	None	N/A	M	72	–	2	0	0	1	12
C3	None	N/A	M	70	–	0	0	0	0	35
C4	None	N/A	F	69	–	0	0	0	0	44
C5	None	N/A	F	49	–	0	0	0	1	29
C6	None	N/A	F	70	–	1	0	0	0	42
C7	None	N/A	M	77	–	2	0	0	0	89
C8	None	N/A	F	75	–	2	0	0	1	55
C9	None	N/A	M	72	–	0	0	0	0	73
C10	None	N/A	M	64	–	0	0	0	0	33
C11	None	N/A	F	78	–	1	0	0	0	45
C12	None	N/A	M	35	–	0	0	0	0	72
C13	None	N/A	M	95	–	2	0	0	0	84
C14	None	N/A	F	61	–	2	0	0	0	28
C15	None	N/A	F	69	–	2	0	0	1	54

*P* pathology, *C* Control, *FTLD-TDP* frontotemporal lobar degeneration with TDP-43 inclusions, *FTLD-U* frontotemporal lobar degeneration with Ubiquitin-only inclusions, *NFT* neurofibrillary tangle, *LP* Lewy pathology, *NP* neuritic plaque, *CERAD* Consortium to establish a registry of Alzheimer’s disease, *PMI* *post-mortem* interval. FTLD-TDP can be categorized into one of four distinct histopathologic patterns of TDP-43 pathology, types A–D. The strength of this histopathologic classification lies in the association between FTLD-TDP subtypes and various clinical and genetic features of disease

### Mitochondrial DNA sequencing

DNA was extracted from each frozen brain sample. The entire mtDNA was amplified with two overlapping long-range PCR amplicons (Primers sequences in Supplementary Table 1, online resource), tagged and indexed applying Illumina Nextera XT reagents and sequenced at high depth with Illumina Miseq v3 chemistry. Each DNA sample was sequenced twice, including independent PCR amplification, library preparation and sequencing.

### Bioinformatic analysis

Sequencing reads were aligned to the human reference genome (hg19 and rCRS NC_012920.1) using MToolBox with an integrated two-step mapping strategy to eliminate potential nuclear DNA of mitochondrial origin (NuMTs) [8]. A probabilistic model Replow was applied to the library-level replicates to enhance the stringency distinguishing artifacts arising during low-heteroplasmy variants calling (HF > 0.5%) [9]. mtDNA variants occurring in homopolymeric regions and at haplogroup defining sites were removed. Annotation and pathogenicity prediction of mtSNVs were performed applying mtoolnote. mtDNA structural changes (deletions, mtDels; and duplications, mtDup) were determined using MitoSAlt which was developed and validated to detect and map mtDNA structural alterations, and to distinguish between deletions and duplications [10]. We applied the following filtering features: cluster threshold = 5 (the minimum number of reads supporting a cluster); break threshold = 5 (the maximum deviation of split read breakpoints can have to be considered within the same cluster); hplimit = 0.005 (detected heteroplasmy threshold); deletion threshold min = 30 (the minimum size of the gap between fragments of a split read for the split read to be considered as potentially spanning a deletion); breakspan = 10 (the minimum number of bases a non-split read must span either side of a breakpoint to be considered in the heteroplasmy count); split distance threshold = 5 (the maximum length of unmapped distance between two fragments of a split read); score threshold = 30 (alignment score cut-off); evalue threshold = 0.00001 (alignment evalue cut-off). Mutational signatures were determined based on single-site pyrimidine substitutions (C > A, C > G, C > T, T > A, T > C, and T > G) and adjacent the 5’ and 3’ bases forming 96 trinucleotide signatures [11]. The substitution rate for each trinucleotide context on the mtDNA light (L) and heavy (H) strands was normalised by the frequency of the trinucleotide context present in the mtDNA reference genome (rCRS) [12], and was compared to the identified 30 nuclear genomic signatures [13].

### Statistical analysis

A multivariate generalised linear model was used to determine which factors correlated with (a) the number of mtSNVs *per* individual; and, (b) the heteroplasmy fraction (HF). An individual’s age, sex, brain region and disease status were included as covariates in the negative binomial regression. A similar model was used to analyse the individual burden, size and HF of mtDels and mtDups. The distribution of mtSNVs in genomic subregions (e.g. coding, non-coding, rRNA and tRNA) between disease groups were compared by Wilcoxon test. The distribution of pathogenicity probability of mtSNVs between disease groups was compared by one-way ANOVA test. To compare the proportion of each mtSNVs signatures between disease groups we performed Fisher’s exact test. The difference in proportions of mis-sense versus synonymous mtSNVs, and in proportions of coding versus non-coding mtSNVs, were compared by Boschloo's exact test. The difference in heteroplasmy level between multi-focal brain regions was examined using one-sample *z*-test, and the difference of cumulative frequency of heteroplasmic mtSNVs between brain regions was computed by two-sample Kolmogorov–Smirnov test.

### Replication

We re-analysed data we published previously where we performed exome sequencing on frozen brain samples from the Medical Research Council Brain Tissue Resource [14]. This included 236 frontotemporal dementia—amyotrophic lateral sclerosis (FTD-ALS) brains (mean age of death of 62.6 years) and 241 aged controls (mean age of death of 72.5 years). The exome sequencing was generated from DNA extracted from the cerebellum in the majority of cases (87.3%). The mtDNA sequence was extracted from off-target exome sequencing reads and variants were called using the bioinformatic pipeline described previously [14], with a detection threshold of VAF > 10% reflecting the limited mtDNA coverage in this study.

## Results

### mtDNA mutation burden with age

**Fig. 1:**
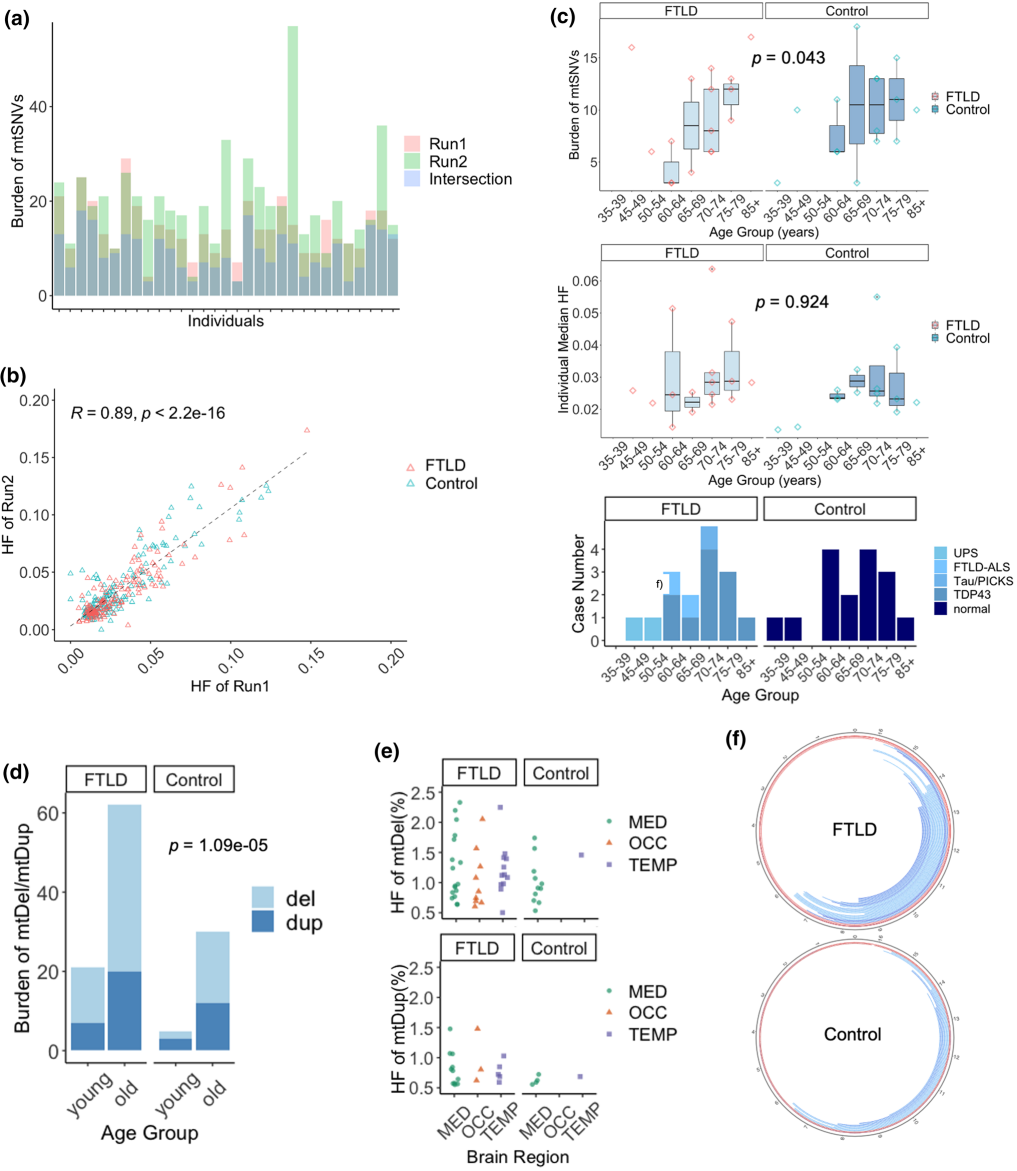
Mitochondrial DNA single nucleotide variants (mtSNVs), deletions (mtDels) and duplications (mtDups) detected in *post mortem* FTLD and age-matched control brains. **a** Individual burden of mtSNVs detected by technical replicates (red: Run 1, green: run 2) and the Replow called intersection (blue). Data from all brain regions was combined. **b** Distribution of heteroplasmy fraction (HF) of mtSNVs between two technical replicates generated following independent library preparations and sequencing. Data from all brain regions was combined. **c** Individual burden and median HF of mtSNVs for different age groups at the time of death. Data from all brain regions was combined. **d** Burden of mtDNA deletions (mtDel) and duplications (mtDup) in young (< = 60 yr) and old (> 60 yr) age groups. Data from all brain regions was combined. **e** HF of the mtDel and mtDup in different brain regions: temporal lobe (TEMP), occipital lobe (OCC) and medulla (MED). **f** The circular plot shows the mtDel (blue) or mtDup (red) segments detected in FTLD and control brains mapped onto the mtDNA molecule. The darkness of each arc represents the HF estimated by MitoSAlt [10]. Data from all brain regions was combined

The mean depth of mtDNA sequencing was 1261 (53–9905) in sequencing run 1 and 1315 (38–6619) in run 2 (Supplementary Fig. 1, online resource). 441 mtSNVs were detected with a variant allele frequency (VAF) > 0.5% in run 1, and 597 in run 2, with a strong correlation between the heteroplasmy fraction (HF, equivalent to the variant allele fraction) in both runs (*R* = 0.89, Fig. 1a, b). To eliminate artifacts introduced by the sequencing, all subsequent analyses focussed on the 290 variants independently detected in the same brain sample by separate PCR amplification, library preparations and sequencing runs. The number of mtSNVs per individual increased with age in both the FTLD and control brains (Negative Binomial Regression Model, *p* = 0.043, Fig. 1c). Brains from older individuals (> 60yrs) contained more mtDels and mtDups than brains from younger individuals (< 60yrs, Negative Binomial Regression Model, *p* = 1.09 × 10^–5^, Fig. 1d, e, Supplementary Fig. 2, online resource). The majority of mtDels mapped to the major arc of mitochondrial genome (Fig. 1f).

### Functional implications of the mtSNVs

**Fig. 2:**
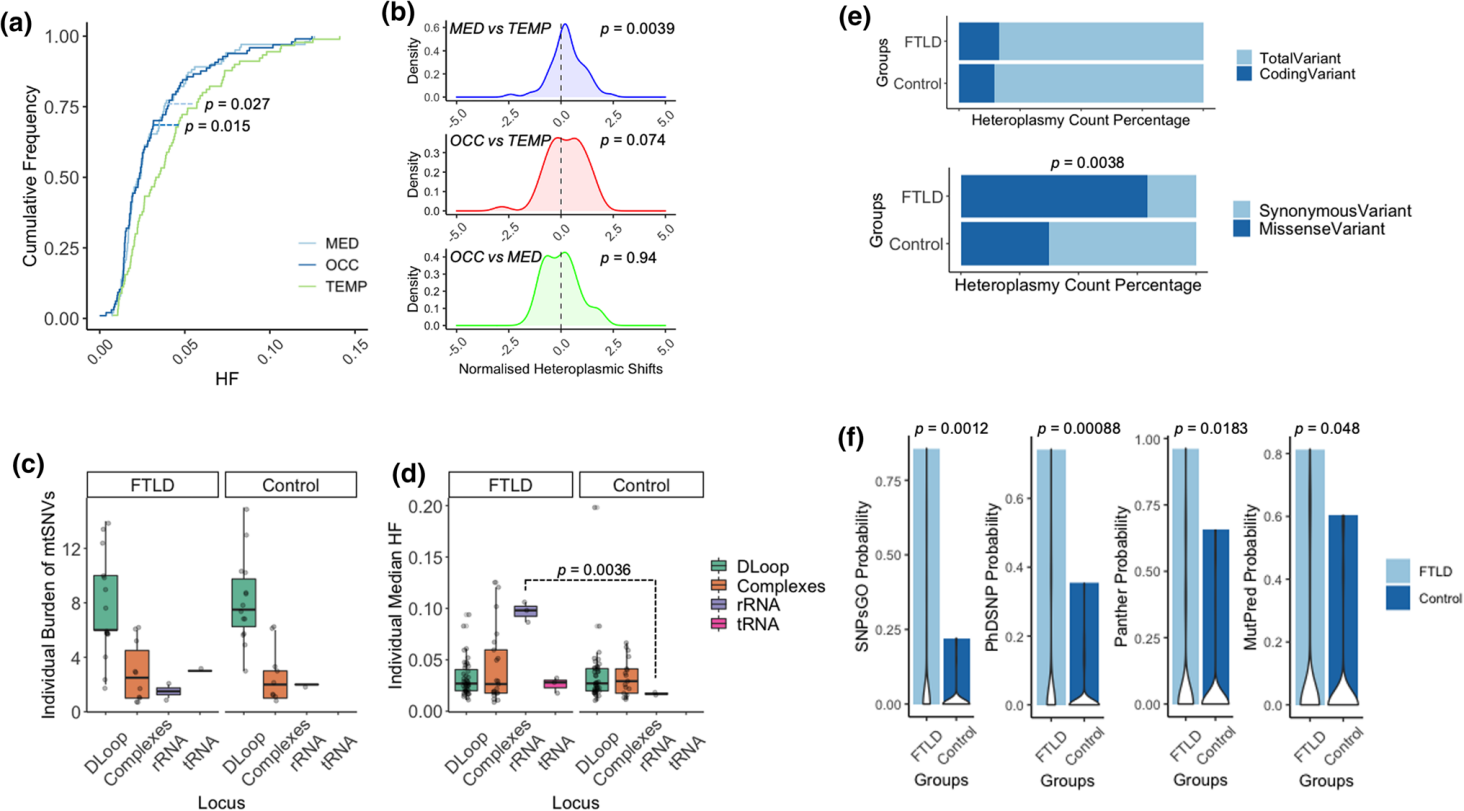
Annotation of the mtDNA single nucleotide variants (mtSNVs) detected in different brain regions. **a** Cumulative heteroplasmy fraction (HF) distribution of mtSNVs detected in temporal lobe (TEMP, light blue), occipital lobe (OCC, dark blue) and Medulla (MED, green) in all cases. **b** Difference in HF between brain region for mtSNVs detected in more than one brain region (multi-focal variants): MED and TEMP (purple), OCC and TEMP (red), and OCC and MED (green). The P-value corresponds to the shift from zero (no difference between the regions). **c** Individual burden of mtSNVs detected in different mtDNA regions: D-Loop, Respiratory chain complex coding regions, rRNA and tRNA genes. Data from all brain regions was combined. **d** HF of mtSNVs detected in the D-Loop, respiratory chain complex genes, rRNA and tRNA genes. Data from all brain regions was combined. **e** The percentage of coding variants and mis-sense variants detected in FTLD and control cases. Data from all brain regions was combined. **f** The pathogenicity prediction of mtSNVs based on SNPsGO, PhDSNP, Panther and MutPred (see methods). Data from all brain regions was combined

Although the total number of mtSNVs in FTLD brains was no different to age-matched controls (Negative Binomial Regression Model, *p* = 0.737 Fig. 1c), in both FTLD and control cases the HF of mtSNVs detected in temporal lobe was higher than mtSNVs detected in medulla (two-sample Kolmogorov–Smirnov test, *p* = 0.0274) and occipital lobe (two-sample Kolmogorov–Smirnov test, *p* = 0.0157) (Fig. 2a). 55 variants present in all three brain regions reached a higher HF in the temporal lobe than the medulla (one-sample *z*-test, *p* = 0.0039) (Fig. 2b).

Next, we annotated the mtSNVs into four categories: (1) non-coding variants in the D-loop; (2) RNA genes; (3) coding variants predicted to alter the amino acid sequence – mis-sense mtSNVs; and (4) coding variants not predicted to alter the amino acid sequence – synonymous mtSNVs. Overall, the greatest mutation burden was seen in the D-loop, followed by protein coding regions, followed by the RNA genes (Fig. 2c). There was no difference in the burden of mtSNVs in different mtDNA regions between FTLD brains and controls. Normalising for the sequence length, the highest mutation frequency per base-pair was in the D-loop (1.58 × 10^–4^), followed by protein coding regions (3.39 × 10^–6^), tRNA (2.99 × 10^–6^) and rRNA (1.54 × 10^–6^). The HF of rRNA variants in FTLD was greater than rRNA variants in controls (*p* = 0.0036, Fig. 2d). Although the overall proportion of coding variants was no different between FTLD brains and controls, a greater proportion of the coding variants were mis-sense mutations in the FTLD cases (Boschloo's exact test, *p* = 0.0038) (Fig. 2e), and the FTLD variants had a greater probability of being pathogenic using multiple prediction tools (one-way ANOVA test, PhDSNP Probability, *p* = 0.00088; SNPsGO Probability, *p* = 0.0012; Panther Probability, *p* = 0.0183; MutPred Probability, *p* = 0.048)(Fig. 2f).

### Origin of the mtSNVs

**Fig. 3:**
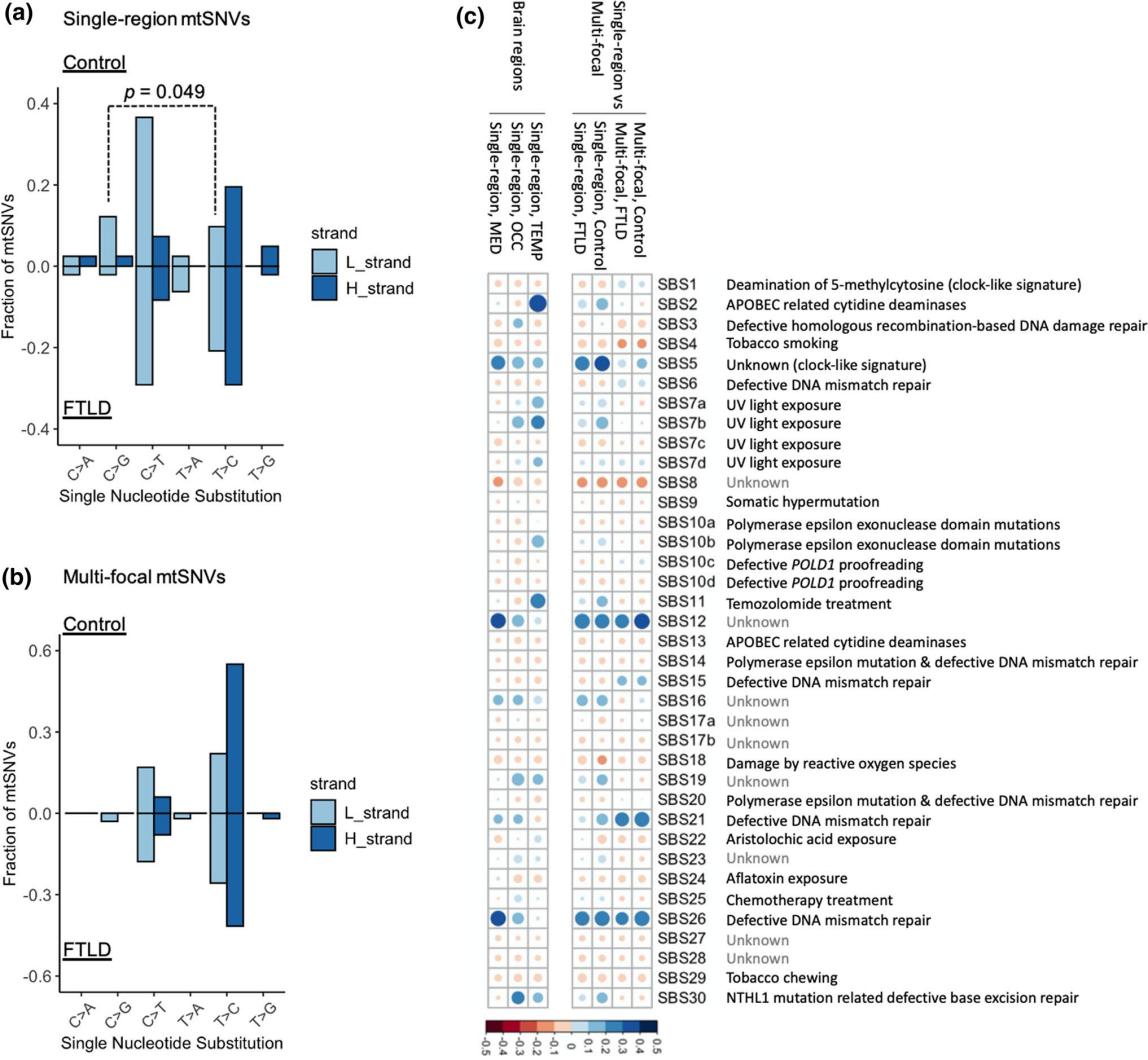
Mechanisms of mtSNV mutations based on the single nucleotide substitution and trinucleotide context. **a** The single nucleotide substitution signature of single-region mtSNVs on both stands (light blue: L strand; dark blue: H strand) between control (top) and FTLD (bottom) cases. Data from all brain regions was combined. **b** The single nucleotide substitution signature of multi-focal mtSNVs on both stands (light blue: L strand; dark blue: H strand) between control (top) and FTLD (bottom) cases. Data from all brain regions was combined. **c** The correlation between trinucleotide mutational signatures observed in the current study with the 30 annotated cancer signatures (see methods). The gradients of circles correspond to correlation *R*^2^ values and the sizes of circles correspond to the *p* values (larger circle with lower *p* values). Left = single region mutations in specific brain regions. Right = single-region vs. multi-focal mutations, data from all brain regions was combined

MtDNA variants detected in multiple brain regions (multi-focal variants) could also have been maternally inherited, or arisen at an early stage in development [15]. However, to be restricted to only one region, single-region variants are likely to have arisen at a later developmental stage. To elucidate their origins, we studied the single and triple nucleotide mutational signatures of single-region and multi-focal mtSNVs separately. There was no difference between FTLD and control brains for the single-nucleotide signature for multi-focal mtSNVs, but for single-region variants there was a higher proportion of T > C substitutions in FTLD, as seen previously in ageing brain (Fig. 3a, b) [16]. For the trinucleotide signatures, the multi-focal mtSNVs showed a similar signature in FTLD and control brains, resembling that seen in defective mis-match repair of the nuclear genome, and an unknown signature, both of which have been seen previously for mtDNA [12]. Single-region mtSNVs showed a ‘clock-like’ signature consistent with age-related base transition mutations, which affected a greater proportion of mtSNVs in FTLD brains. Overall, mtSNVs in the temporal lobe showed a brain region-specific signature characterised by cytosine mutations generated during DNA replication, consistent with spontaneous deamination of 5-methyl-cytosine as observed in single neurons (Fig. 3c) [17].

### Replication

Analysis of the previously published data [14] confirmed the higher mutation frequency of rRNA coding mtSNVs, and the greater proportion of mis-sense variants in FTD-ALS brains compared to controls (Boschloo's exact test, *p* = 0.029) (Supplementary Fig. 3).

## Discussion

Our observations provide two independent strands of evidence supporting a role for mtDNA mutations in the regional pathology of FTLD. First, we show that the temporal lobe of older individuals harbours more mtSNVs at a higher heteroplasmy level than other brain regions. Second, in FTLD, a greater proportion of the mtSNVs were predicted to have functional consequences—either directly on conserved amino acids in essential respiratory chain proteins, or on RNA genes involved in intra-mitochondrial protein synthesis.

Previous studies of Parkinson’s and Alzheimer’s disease have described an association with mtDNA mutations, but it has not been possible to separate cause from effect. Our findings indicate that the age-related accumulation of mtSNVs is not directly influenced by FTLD because we saw the same mtSNV burden with age in brains from healthy controls. However, the FTLD brains contained more mtSNVs predicted to affect mitochondrial function. There is an emerging literature linking secondary mutations of nuclear DNA with the pathophysiology of FTLD. For example, TDP-43 dysfunction may contribute to mtDNA mutagenesis through defective double-strand break repair [18]. In keeping with this, we observed a higher burden of mtDels and mtDups in brains from older individuals with FTLD, with the majority of the mapped mtDNA deletions removing key peptide-encoding and RNA genes crucial for oxidative phosphorylation. However, the disease mechanisms of FTLD mechanisms are unlikely to preferentially target mtDNA nucleotides that cause amino acid substitutions, nor have a predilection for specific mtDNA genes that spares other regions of the mitochondrial genome. Thus, the specific pattern of variants we observed in FTLD temporal lobe points towards a causal role. This will require independent validation.

Although the majority of mtSNVs and mtDels were detected at low heteroplasmy levels (< 10%), recent single-cell analyses have shown that even low-level heteroplasmies (< 5%) can alter the transcript levels of nuclear genes involved in ATP synthesis and key cellular processes such as telomere maintenance which have also been implicated in aging and age-related diseases [12]. The results we present here are based on high-depth sequencing of DNA extracted from bulk tissue, so the heteroplasmy values represent an average across millions of cells, of different types. Heteroplasmy levels can differ markedly between cells, making it likely that some individuals neurons and glia also contain very high levels of specific variants which directly affect mitochondrial function. Although this may only affect a small proportion of cells, the mtDNA mutations may contribute to the clinical features because the affected cells disrupt neuronal networks, or because they act in parallel to other disease mechanisms in different cells.

This work also provides insight into the origin of mtSNVs. As a conservative estimate, the number of cells in each brain sample we studied was ~ 3 × 10^5^ [15]. Given our mutation detection threshold of > 0.5%, each mtSNV must have been present in a very large number of cells, or it would not have been detected, and must therefore have arisen early in brain development. Mutations arising at a late stage would be limited to one or a few cells, and thus not be detectable by our methods. We observed distinct mutational signatures for mtSNVs present in multiple brain regions (multi-focal) to those only found in a single brain region. This implies a different mechanism linked to the origin of the mutations, with the clock-like or ‘ageing’ signature only seen in single-region variants occurring later in development. This was not seen in the multi-focal mtSNVs which were likely inherited or occurring early in brain development. Similar mutational signatures seen in FTLD and control brains supports our conclusion that the underlying mtDNA mutation rate is the same in health and disease, but if the mtSNVs affect mitochondrial function, they are more likely to be found in FTLD brains than controls and at higher heteroplasmy levels.

Measuring low-frequency DNA variants is technically challenging because of the possibility of artifacts introduced by the technique. To minimise this possibility we performed stringent, validated quality controls in our bioinformatic pipeline, excluding known systematic errors arising through repetitive DNA sequences or nuclear-encoded mitochondrial sequences (NuMTs). Of critical importance, we independently sequenced the original DNA sample twice. Although doubling the cost, this provided us with reassurance that the mtSNVs we detected with present in the original brain tissue and not an artifact. Finally, to provide independent validation of our findings, we re-analysed published data [14]. Despite there being several technical differences between the two studies, the replication analysis confirmed the higher mutation frequency of rRNA coding mtSNVs, and the greater proportion of mis-sense variants in FTD-ALS brains compared to controls. The technical differences between the two studies included the combined analysis of FTD-ALS in the published study compared to FTLD in the current study; the tissues used for DNA extraction (cerebellum in 87.3% of the published study compared to temporal lobe, occipital lobe and medulla in the current study); the mean sequencing depth (289 in the published data, and 1261 of run 1 and 1315 of run 2 of the current study); and thus, different heteroplasmy detection thresholds (VAF > 10% in the published study).

Several genetic causes of FTLD are converging on overlapping mitochondrial mechanisms with downstream effects on ATP synthesis, bioenergetics, and calcium signalling (*C9ORF72*); mitochondrial transcription (*SQSTM1, FUS*), protein import (*UBQLN2*), and respiratory complex assembly (*C9ORF72*). Here we provide evidence that heteroplasmic mtSNVs are additionally important in the pathogenesis of FTLD. Mitochondria are metabolic hubs, integrating many essential cellular processes beyond oxidative phosphorylation. The mtSNVs we have identified can influence the metabolic hubs directly, interacting with the additional mechanisms listed above, and thereby providing mechanism to exacerbate the regional pathology which underpins the clinical features of the disorder.

## Supplementary Information

Below is the link to the electronic supplementary material.

Supplementary file1 (DOCX 2801 KB)
