# Supplementary material for: Heteroplasmic mitochondrial DNA mutations in frontotemporal lobar degeneration
Source: Acta Neuropathol. 2022 Apr 30;143(6):687–95. doi: 10.1007/s00401-022-02423-6 (PMC9107417; doi:10.1007/s00401-022-02423-6)
Supplement: Supplementary file 1 — Supplementary file1 (DOCX 2801 KB) [file 401_2022_2423_MOESM1_ESM.docx]

**Supplementary Table and Figures**

**Supplementary Table 1**

Primer sequences

| **Sequence Name** | **Sequence** |
| --- | --- |
| MTL1_F | CCCTCTCTCCTACTCCTG |
| MTL1_R | CAGGTGGTCAAGTATTTATGG |
| MTL2_F | CATCTTGCCCTTCATTATTGC |
| MTL2_R | GGCAGGATAGTTCAGACG |

**Supplementary Figure 1**

The distribution of sequencing depth across mitochondrial genome in FTLD cases replicate 1 (top left), FTLD cases replicate 2 (top right), control replicate 1 (bottom left) and control replicate 2 (bottom right).

**Supplementary Figure 2**

Distribution of mtDNA deletions (mtDel) and duplications (mtDup) between disease conditions: Burden in different brain regions and age groups (top left); Distribution of HF in different brain regions and age groups (top right); HF in different age groups (bottom left); size of mtDel or mtDup in different age groups (bottom right).

**Supplementary Figure 3**

Analysis of published data from the MRC Brain Tissue Resource^13^. MtDNA sequences were derived from off-target exome reads in 236 frontotemporal dementia - amyotrophic lateral sclerosis (FTD-ALS) brains (mean age of death of 62.6 years) 241 aged controls (mean age of death of 72.5 years), showing (a) the burden of mtSNVs normalised by case number in control and ALS-FTD groups; and (b) the percentage of different types of mtSNVs in control and ALS-FTD groups. This analysis replicated the main findings published here, despite there being three technical differences between the current study and the published study. The mean depth of coverage was 289 in the published data, and 1261 of run 1 and 1315 of run 2 of the current study. In the published study, the DNA was extracted from the cerebellum in majority cases (87.3%), instead of 3 brains regions including temporal lobe, occipital lobe and medulla of the current study. As a result, there was insufficient depth to examine mtDNA mutations with a variant allele fraction (VAF) <10%, to check for mtDNA rearrangements, and study the temporal lobe specifically. Finally, we were unable to separate FTD from ALS cases, which were considered as a single group.
